# Description of the immature stages of the planthopper *Lacertinella australis* (Hemiptera: Delphacidae)

**DOI:** 10.1093/jis/14.1.113

**Published:** 2014-08-12

**Authors:** M. F. Rossi Batiz, A. M. Marino de Remes Lenicov, Henry Hagedorn

**Affiliations:** 1 División Entomología, Facultad de Ciencias Naturales y Museo, Universidad Nacional de La Plata. Paseo del Bosque S/Nº (B1900FWA), La Plata, Buenos Aires, Argentina; 2 Scientific Research Committee of the Province of Buenos Aires (CIC), Argentina

**Keywords:** Argentina, crop pest, nymphal development

## Abstract

The five immature stages of the planthopper *Lacertinella australis* (Remes Lenicov and Rossi Batiz) (Hemiptera: Delphacidae: Saccharosydnini) are described and illustrated. The main characters that allowed us to distinguish the various stages were body size, number of tarsomeres and metatibial spines, and number of teeth on the spur. New biological data based on laboratory rearing and field observations showed that *L. australis* can carry out its biological cycle successfully on the graminaceous pampas grass ( *Cortaderia* spp. Stapf (Poales: Poaceae)). In addition, the efficient rearing in captivity, the high survivorship registered, and overwintering only on this host plant suggests that *L. australis* is a potential biocontrol agent of this invasive graminaceous weed. This study provides information about the immature stages *,* including a key for their identification, based on laboratory reared specimens and field observations.

Resumen

Se describen e ilustran las cinco etapas inmaduras de la especie de Saccharisydnini *Lacertinella australis* (Remes Lenicov and Rossi Batiz) (Hemiptera: Delphacidae). Los principales caracteres que permitieron distinguir las diferentes etapas fueron: tamaño corporal, número de espinas en los tarsómeros y metatibia y número de dientes en el espolón tibial. Nuevos datos biológicos, basados en la cría de laboratorio y observaciones de campo, mostraron que *L. australis* puede realizar su ciclo biológico exitosamente en la graminácea cortadera ( *Cortaderia* spp. Stapf (Poales: Poaceae)). Además, la eficiente crianza en cautive-rio y la alta supervivencia registrada en esta planta hospedera, sugieren que *L. australis* podría ser usada como un potencial agente de control biológico de esta maleza invasora. Este estudio proporciona informa-ción sobre las etapas inmaduras, incluyendo una clave para su identificación, basada en individuos provenientes de la cría de laboratorio y de campo.

## Introduction

The genus *Lacertinella* (Remes Lenicov and Rossi Batiz, 2011) (Hemiptera: Delphacidae) includes at present only one species, *Lacertinella australis* ( Rossi Batiz and Remes Lenicov 2011b , 2012), which is widespread in Argentina, breeding frequently on pampas grass ( *Cortaderia* spp. Stapf (Poales: Poaceae)). Other graminaceous have been recorded as host plants, including rye ( *Secale cereale* ), rice ( *Oriza sativa* ), and garlic ( *Allium sativum* ), in northern and central agricultural areas of Argentina (Rossi Batiz and Remes Lenicov 2011).

According to Vibrans (2007) , pampas grass is a South American native perennial graminaceous plant with high tolerance to drought and alkaline, acidic, clayey, and sandy soils; it is highly adaptable, growing in a wide range of environments and climates. It is used as a wind barrier between crops and as a nitrogen-fixing plant in agricultural soil. It was introduced in Europe and North America as an ornamental and forage plant and has been carried to areas where it is now a pest because it disperses so easily. Thus, in California, Hawaii, Australia, or northwest of the Iberian Peninsula, it is an invasive species along irrigation canals in vacant land and plantations (e.g., pine). In New Zealand and South Africa, its sale and transportation are prohibited to prevent its establishment. In other parts of the world, it also invades commercial plantations. Because of its sharp-edged leaves, it produces injuries and allergies in human skin.

Nothing is known about of the fauna associated with this weed, which could be useful as potential biological control agents in regions where pampas grass is considered a pest.

Two species of native Saccharosydnini are closely associated with this plant in Argenti na: *Saccharosydne subandina* Remes Lenicov and Rossi Batiz and the recently described *L. australis* ( Rossi Batiz and Remes Lenicov 2011b , 2012). There is lack of biological and ecological knowledge about this tribe. At pre sent, information is only available about the immature stages and life cycle for two species: *Saccharosydne saccharivora* (Westwood) from Venezuela, Jamaica, Belize, and Cuba ( Guagliumi 1953 ; Metcalfe 1969 , 1972), and *S. subandina* from Argentina ( Rossi Batiz and Remes Lenicov 2011b ).

In this paper, we describe the immature stages of *L. australis* based on laboratory-reared specimens, and we include a key for their separation. Biological information from field observations is also included.

## Materials and Methods

Specimens collected from La Plata, Buenos Aires, between 2011 and 2012 were used to maintain a laboratory colony on pampas grass in order to describe stages of development, oviposition scars, damage to the plants, and eventual natural enemies. For one year, insects were reared on two plants in outdoor conditions under the protection of a roof. Plants were in pots with soil, covered by voile, and irrigated periodically with water.

### Morphological studies

The description of each stage is based on 24hr hatched nymphs from the laboratory colony. Specimens were anesthetized by freezing to register coloration, cleared in 10% KOH solution, and fixed in glycerine for microscopic examination and illustration.

The first and fifth instars are described in detail; only major changes are highlighted in the intermediate instars. The nomenclature for carination and arrangement of pits follows Yang and Yeh (1994) . Drawings were made using a stereoscopic microscope with a camera lucida.

The measurements derive from 10 specimens of each stage and are given in millimeters. The egg measurements were taken in the redeye instar of development. Measurements are expressed as mean ± standard error (SE) (Table 1).

**Table 1.**
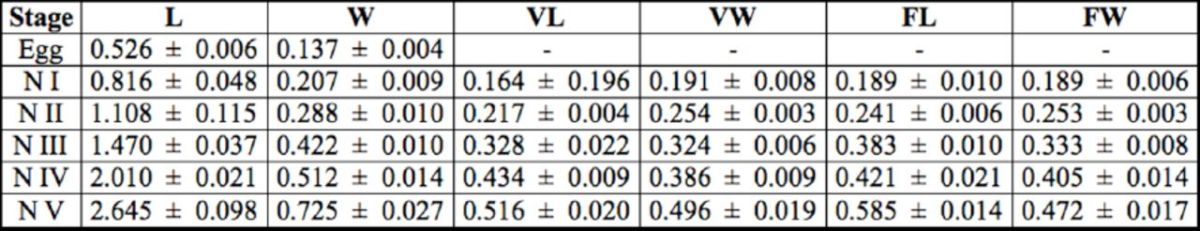
Measurements (mean ± SE, mm) of the immature stages of *Lacertinella australis* .

Measurements: body length (L); body width (W); vertex length (VL); vertex width (VW); frons length (FL); frons width (FW).

To check the stability of the characters, the specimens from the laboratory colony were compared with individuals collected in different localities of Argentina in 2008, 2011, and 2012.

## Results

### Eggs( Fig. 1 )

Ellipsoidal with cephalic apex and opposite end rounded; ventral surface slightly concave, dorsal convex. Color milky white when laid, turning white yellowish before hatching. Chorion translucent, smooth.

**Figures 1–6.**
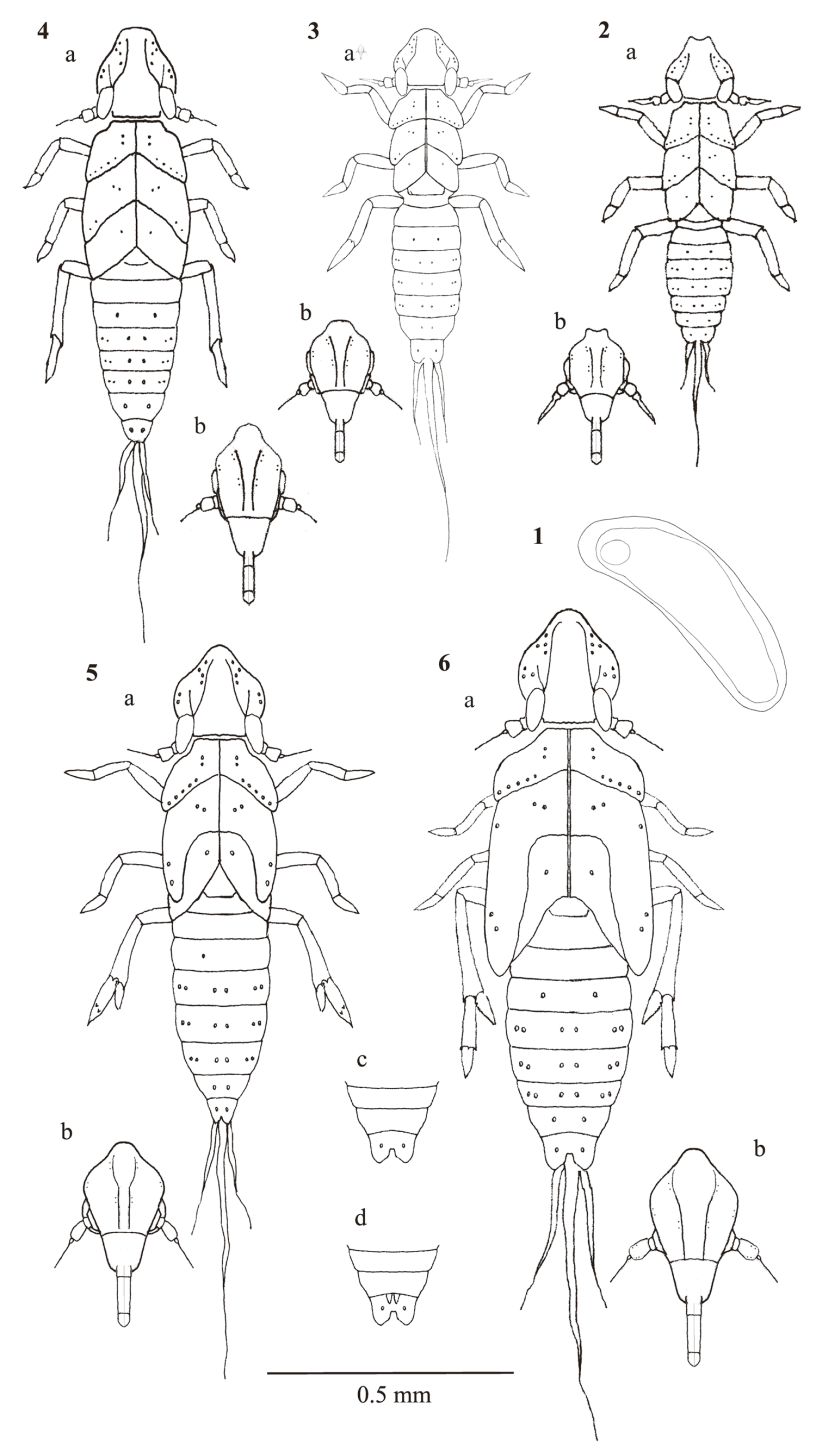
Immature stages of *Lacertinella australis* : 1. Egg; 2. First instar (a) nymph, (b) frons; 3. Second instar (a) nymph, (b)frons; 4. Third instar (a) nymph, (b) frons; 5. Fourth instar (a) nymph, (b) frons; 6. Fifth instar (a) nymph, (b) frons, (c) male and (d) female ventral abdominal view. High quality figures are available online.

### First instar( Fig. 2 )

General color uniform light green; eyes orange. Brown on pits, claws, and leg spinulation with a distinctive white layer of wax covering the whole body; numerous fine

filaments extending posteriorly from the abdomen, grouped in long bundles tails, some as long as the whole body. Elongated subcylin-frical in form, widest across head and mesothorax. Vertex 1.16× as long as the width at base, projecting beyond eyes 2/3 its length; anterior margin bilobed, slightly depressed in middle, posterior margin straight, lateral margins carinated; submedian carinae arising near anterior margin of eyes, near to lateral carinae, both not reaching apex. Frons as long as wide, widest at middle length; with two submedian carinae close to each other, diverging toward the base, where they become obscure, delimiting a moderately inflated snout-like area at apical third; in frontal view, lateral carinae diverging from base to median third, then gradually converging from anterior margin of eyes toward apex; interfrons much narrower than laterofrons. Clypeus 1.25× longer than broad. Rostrum three-segmented, almost reaching metacoxae; segment I almost completely obscured by anteclypeus; II and III subequal. Antennae three-segmented; segments I and II wider than long; II 2× longer than I; III bulbous basally, ending in a bristle reaching procoxae. Thoracic nota divided into two pairs of plates by longitudinal mid-dorsal line. Pronotal plates subtrapezoidal, lateral carinae divergent, slightly convex toward posterior margin. Mesonotum and metanotum plates subrectangular, posterior margin convex, metanotum subtrapezoidal, longer laterally. Legs subcylindrical; metafemur slightly longer than metatibiae; metatibiae unarmed laterally, bearing apical row of five brown-tipped spines with the internal one longest; metatarsomeres equal in length, metatarsomere I bearing apical row of four brown-tipped spines; metatarsomere II undivided, two claws. Abdomen 11-segmented, subcylindrical, getting wider at level of segments IV and V.

Arrangement of pits (on each side): *Head:* four pits between submedian and lateral carinae of vertex; two before eyes between lateral carinae of vertex and frons; six on frons, upper and lower pairs nearer submedian carinae and median pair nearer lateral carinae. *Thorax:* seven pits on pronotum: two between midline and lateral carinae, five lined near posterior margin outside lateral carinae; four on mesonotum wingpads, one between midline and lateral carinae, one outside lateral carinae, two near posterolateral angles; one on metanotum, near posterior margin on each side of lateral carinae. *Abdomen:* one submedian pit on IV segment; three on tergum of segments V-VII (one submedian and two lateral on each side of segment); one on segments VIII; three on segment IX, two on dorsal and one on ventral surfaces.

### Second instar( Fig. 3 )

Coloration as in previous instar. Vertex, frons, and carinae as in previous instar. Antennal segment II as long as wide. Posterior margins of mesonotum slightly overlaping metanotum. Metatibia 1.5x the length of metafemur; metatarsi I slightly longer than II, bearing apical row of five brown-tipped spines.

Arrangement of pits as for first instar.

### Third instar( Fig. 4 )

Coloration as in previous instars. Vertex and frons as long as wide, submedian carinae of vertex reaching apex. Antennal segment II 1.5x the I length. Mesonotal wingpads reaching basal half of metanotum. Wingpads posterolaterally slightly angular. Metatibiae bearing apical row of five spines + moveable subconical spur; spur half as long as metatarsomere, without marginal teeth. Metatarsi two-segmented; metatarsomere I 1.5x longer than II, bearing apical row of six brown-tipped spines with external longest; metatarsomere II with an obscure transversal sulcus in the middle.

Arrangement of pits as in former instars.

### Fourth instar( Fig. 5 )

Coloration similar to former instar but darker. Vertex and frons longer than wide. Antennal segment II twice as long as I. Mesonotal wingpads covering >2/3 of metanotal plates laterally; metanotal wingpads extending to third abdominal segment. Metatibiae as long as metafemur, bearing seven apical spines + spur slightly longer than metatarsomere II+III, bearing apical tooth and 4-6 marginal teeth; matatarsi with tarsomere I bearing an apical row of seven spines (2+5); tarsomere II with two spines near middle of partially subdivided tarsomere.

Arrangement of pits as in former instars.

### Fifth instar( Fig. 6 )

Coloration similar to former instars, but darker. Body flattened. Head longer than previous instars, protruding beyond level of eyes almost 2/3 of vertex length. Lateral carinae of vertex reaching lateral carinae of frons; sub-median carinae attaining basal margin of vertex laterally; basal compartment 1.5x longer than at base. Y-shaped carinae obscure. Eyes 2xlonger than wide at widest portion. Frons 1.24x longer in middle line than wide at widest portion, submedian carinae reaching frontoclipeal suture, subparallel on apical third of frons and diverging toward apex. Each laterofrons at widest portion 1.33× wider than interfrons at widest portion. Antennae: I and II segments almost as long as wide; II 2× longer than I. Rostrum three segmented: basal and apical segments subequal in length; subapical 1.5× the length of the others, pigmented at apex. Mesonotal wingpads almost as long as metanotal ones, reaching basal half of IV abdominal segment. Metatibia bearing two small black-tipped spines on outer margin, one near base, other nearly mid-length, 1.14× longer than metafemur, bearing apical row of seven (2+5) brown-tipped spines. Metatarsomere I three-segmented, tarsomere I bearing ventral apical row of eight brown-tipped spines (2+6) and four ventral brown-tipped spines, respectively. Metatarsi almost as long as metatibia. Spur foliate, 3× longer than broad at broadest portion, as long as 2/3 the length of metatarsite I, with 13‒14 marginal brown-tipped teeth of similar size. Spinal formula of hind leg: 7(5+2)‒8(6+2)‒4. Between abdominal segments VIII and IX ,it can be distinguished the growing dorsal valves in female nymphs ( Fig. 6 , c and d).

Arrangement of pits (on each side): *Head* : three pits between eyes and lateral carinae of vertex; four pits between lateral carina and submedian carinae of vertex; six on frons, upper and lower pairs nearer submedian carinae and median pair nearer lateral carinae. *Antennae* : four pits. *Thorax* : seven pits on pronotum: two, between midline and lateral carinae, five on posterior margin outside lateral carinae; five on mesonotum wingpads, one near anterio-lateral angles, one near posterior margin on each side of lateral carinae, and one on posterolateral angles; one on metanotum: outside lateral carinae. *Abdomen* : one on tergum of segment IV; three on segments V‒VII; two on segments VIII and IX.

### Key to the nymphal instars of *L. australis:*

1-Vertex longer than wide; metatarsi three-segmented; if two-segmented, then with two spines in the middle of tarsomere II; metatibiae with two lateral spines…….……………. 2

*1-* Vertex as long as wide; metatarsi two-segmented; metatibiae with two lateral spines or without any spines; tarsomere II without spines……….………………..……….……. 3

2-Metatibia with six apical spines + spur bearing 13-14 submarginal teeth; metatarsi I with eight apical spines; metatarsi II bearing four ventral spines; mesonotal wingpads laterally overlapping the metanotal wingpads and attaining fourth abdominal segment ( Fig. 6 )……………………………....…fifth instar 2’ *—* Metatibia with six apical spines + spur bearing 4-6 submarginal teeth; metatarsi I with apical row of seven spines; tarsomere II with two spines near middle of partially subdivided tarsomere; mesonotal wingpads covering >2/3 of metanotal plates laterally; metanotal wingpads extending to third abdominal segment ( Fig. 5 ) ……..fourth instar

3-Metatibiae with two lateral spines on shaft and six apical spines; metatibial spur without marginal teeth; metatarsomere I with apical row of six spines; metatarsomere II with an obscure transversal sulcus in the middle; mesonotal wingpads reaching basal half of metanotum ( Fig. 4 ) ...……………third instar 3’-Metatibiae without lateral spines and three or four apical spines; metatibial spur marginal without teeth; metatarsi II undivided…..……4

4-Metatibiae with four apical spines plus movable spur with apical tooth; metatarso mere I with apical row of five spines; notal plates of meso and metathorax slightly larger than prothorax ( Fig. 3 )………...second instar 4’-Metatibiae with apical row of four spines, with internal one longest; metatarsomere I with apical row of four spines; notal plates of thorax similar in size ( Fig. 2 )……..first instar

### Other specimens examined

Argentina: 1 nymph V, Huerta Grande, Córdoba, hand captured on pampas grass, 29-VII-2008. Virla, leg.; 1 nymph V, Cerro San Javier, Tucumán, hand captured on pampas grass, 20-V-2008. Virla, leg.; 6 nymphs I, 1 nymph III, 1 nymph IV and 3 nymphs V, Mir-ador del Lago, Córdoba, captured on pampas grass, 29-VI-2008. Virla, leg.; 2 nymphs I, 3 nymphs II, 3 nymphs III, 2 nymphs IV, 3 nymphs V, La Plata, Buenos Aires, hand-captured on pampas grass, 08-IV-2011. Rossi Batiz leg; 3 nymphs I, 3 nymphs II, 4 nymphs III, 3 nymphs IV, 4 nymphs V, City Bell, La Plata, Buenos Aires, hand-captured on pampas grass, 29-IV-, 26-X-2011, 04-III- and 08-IV-2012. Rossi Batiz leg.; 3 nymphs IV, 1 nymph V, La Plata, Buenos Aires, hand-captured on pampas grass, 06/V/2011. Rossi Batiz leg.

### Biological aspects

Mating occurs on abaxial surface of the leaf, and the eggs are laid along the veins inside the tissues of pampas grass, which is the single host for breeding currently registered. Ovipositional scars are recognizable along the leaves because they are covered with a white wax contrasting with the green color of the plant.

The damage caused to the plant was similar to that described by Guagliumi (1953) for *S. saccharivora,* and it consisted of injuries to the tissues during feeding and ovipositing that resulted in a shift to an orange color and the infection caused by a black fungus growing on the wax excreted by the nymphs and females, which may prevent normal respiration and photosynthesis. Large quantities of eggs and wet ambient conditions also can cause the putrefaction of the plants.

*S. saccharivora* also produces severe damage by transmitting the sugarcane yellow leaf phytoplasma and secondary development of Fumagina (sooty mold) ( Guagliumi 1953 , Arocha et al. 2005 ) on the graminaceous sugarcane *(Saccharum officinarum* L.) in Tropical America. Although *L. australis* was associated with garlic crops infected with the 16SrIII (x-disease) group ( Galdeano et al. 2004 , 2009; Rossi Batiz et al. 2007), it has not been implicated as a vector.

Taking into account the behavioral and morphological similarities of *S. saccharivora* and *L. australis,* the latter deserves more research because of its potential capacity as a biological control agent for pampas grass.

An unidentified species of Strepsiptera (Elenchidae) was found parasitizing adults of *L. australis* and is the only known natural enemy ( Remes Lenicov and Rossi Batiz 2010 ).

### Remarks

Nymphs share common biological features with *S. subandina,* such as the presence of a layer and abdominal filaments of wax; food preferences; same host for breeding; crowding of adults and nymphs around the masses of eggs in any sector of the plant; eggs covered with wax; and type of damage to the host plant (including clorotic orange marks on the injuries produced by feeding and ovipositing and the wax that is invaded by the black fungus).

Frequently, the pampas grass observed in Argentina was highly colonized by one or both species; *L. australis* is more common in the eastern provinces (Entre Ríos, Córdoba, La Pampa, and Buenos Aires), and *S. subandina* is more abundant in the western provinces (Ju-juy, Tucumán, La Rioja, San Juan, Mendoza, Neuquén, and Río Negro) ( Remes Lenicov and Rossi Batiz 2010 , Rossi Batiz and Remes Lenicov 2011a ). Both species had their highest population densities during spring (Rossi Batiz, unpublished data). Nymphs of each species can be differentiated from the second instars by taking into account the following features.

*S. subandina:* Nymphs II, III, IV, and V: light yellowish color, with four lateral orange stripes along dorsal surface of body; eyes yellow. Nymph V: VL represents 1/9 of total length.

*L. australis:* Nymphs II, III, and IV: uniform green yellowish color, without stripes on the surface of body; eyes orange. Nymph V: vertex length represents 1/5 of total length.
